# Identifying Potential Drug Targets for Membranous Nephropathy Through an Analysis of Mendelian Randomization

**DOI:** 10.7759/cureus.100413

**Published:** 2025-12-30

**Authors:** Yasin Abdi Saed, Ahmed Ibrahim Mohamed, Mohamed Mohamoud Adan, Muhammad Attique, Noor Sarfraz

**Affiliations:** 1 Department of Medicine, International College of Medicine and Pharmacy, Changsha Medical University, Changsha, CHN; 2 Health Science Center, Xi'an Jiaotong University, Xi'an, CHN; 3 Department of Internal Medicine, The First Affiliated Hospital of Xi'an Jiaotong University, Xi'an, CHN; 4 Department of Urology, Sir Run Run Shaw Hospital, Zhejiang University School of Medicine, Hangzhou, CHN

**Keywords:** circulating plasma protein, drug repositioning, membranous nephropathy, mendelian randomization, retrospective

## Abstract

Membranous nephropathy (MN) is the most common cause of adult nephrotic syndrome. Current treatments rely heavily on immunosuppressants; however, some patients do not achieve the desired therapeutic effect. Therefore, the identification of new drug targets and the development of novel medications are of urgent importance. In this study, we collected protein quantitative trait loci (pQTLs) for 734 circulating plasma proteins (CPPs) from previous research. Using principles of Mendelian genetics, we conducted a Mendelian randomization (MR) analysis using three inferential methods: the Wald ratio, inverse-variance weighted (IVW), and MR-Egger.

After assessing heterogeneity, we identified 16 CPPs that were positively correlated with the occurrence of MN and 14 CPPs that were negatively correlated with MN. Enrichment analysis showed that these CPPs are primarily involved in oxidative stress and inflammation pathways, which are closely related to the development and progression of MN. Subsequently, using the Drug Signatures Database (DsigDB), we predicted potential drugs that might interact with these CPPs. Finally, we found that curcumin, a natural compound known for its antioxidant, anti-inflammatory, and immunomodulatory properties, could be a potential therapeutic molecule for the treatment of MN.

This study aimed to systematically screen CPPs for causal associations with MN risk using MR, characterize the biological pathways of implicated proteins, and nominate potential therapeutic agents via computational drug repurposing analysis.

## Introduction

Membranous nephropathy (MN) is a common cause of nephrotic syndrome, particularly affecting middle-aged adults of European ancestry. The primary clinical signs are nephrotic syndrome with proteinuria, edema, and hypoproteinemia [1]. Compared to other common forms of autoimmune nephropathy, such as IgA nephropathy and lupus nephritis, the occurrence of hematuria in MN is uncommon and often negligible. Furthermore, some MN patients experience spontaneous remission [2]. Although approximately 30% of patients recover naturally, a similar proportion of non-responsive patients may experience renal failure within 15 years despite receiving immunotherapy [3]. The study by Troyanov et al. revealed that the rate of renal survival was 100% for patients who achieved complete remission and 70% for those who experienced partial remission. This suggests that with proper treatment and timely intervention, the risk of renal failure can be significantly reduced or nearly eliminated [4]. While previous omics studies have provided valuable insights into the associations between MN and circulating plasma proteins (CPPs), the focus on correlation analyses has hindered efforts to establish a causal relationship. This limitation has significant implications for drug development.

In this study, we employed Mendelian randomization (MR) analysis to address this challenge. This framework allowed for robust causal validation, capitalizing on the MR paradigm's inherent resistance to confounding factors [5]. We collected genome-wide association study (GWAS) data for CPPs and MN. After conducting our analysis, we identified a significant causal relationship between CPPs and MN. Based on these findings, we identified drug molecules from the database as potential candidates for the treatment of MN.

To address the gap in causal evidence for therapeutic targets in MN, we performed a multi-stage analysis. The primary objective was to identify CPPs with a putative causal relationship with MN risk using a multi-method MR framework. Proteins surviving robust sensitivity analyses were then carried forward to address secondary objectives: to elucidate their involved biological pathways and protein-protein interaction (PPI) networks, and to computationally screen for existing drugs or compounds that target these proteins, thereby nominating candidates for drug repurposing in MN.

## Materials and methods

Data sources

The study was conducted at The First Affiliated Hospital of Changsha Medical University, Changsha, China. For the summary-level GWAS data pertaining to CPPs, we selected a recent study conducted by Zhang et al. [6]. In their research, they amalgamated data from five large-scale plasma protein GWAS studies and ultimately curated protein quantitative trait loci (pQTL) data for analysis, encompassing 734 CPPs. As for MN, we sourced the relevant GWAS data from the IEU Open GWAS database [7]. Given that the population for pQTL data research was of European descent, we exclusively selected European ancestry MN-related data with the accession number: ebi-a-GCST010005.

Mendelian randomization

Before conducting MR analysis, we performed single-nucleotide polymorphism (SNP) screening on the 734 proteins to ensure adherence to the fundamental assumptions of MR. To achieve this, we used the PhenoScanner platform (MRC Integrative Epidemiology Unit, Bristol, United Kingdom) to identify and exclude SNPs potentially associated with conditions such as MN and hypertension [8]. Since the majority of CPPs had only one available SNP, we primarily employed three methods: the Wald ratio, inverse-variance weighted (IVW), and MR-Egger, to assess the causal relationship between CPPs and MN [9].

Firstly, when only one available SNP served as the instrumental variable (IV), we employed the Wald ratio method, which provides highly accurate estimates when the IV is a single SNP. When there was more than one IV, we relied on the IVW method as the primary reference [10]. This method assumes an intercept of 0 and offers the most precise estimates in the absence of pleiotropy. Conversely, MR-Egger permits the existence of an intercept and provides more accurate results when there is heterogeneity [11].

Simultaneously, we conducted sensitivity analyses by employing the Q-test to detect SNP heterogeneity, flagging the presence of heterogeneity when p-values were less than 0.05. We also performed MR-Egger intercept tests. If results indicated the presence of pleiotropy, we further corrected for potentially confounded SNPs to ensure the accuracy of the final results. As this study is exploratory in nature, we refrained from adjusting p-values, deeming a threshold of p < 0.05 as significant to avoid overlooking potential positive findings [12].

Enrichment analysis and PPI network

For the CPPs that demonstrated a significant causal relationship with MN, we conducted enrichment analysis using the clusterProfiler package (R package, Bioconductor Project (Yu Lab), Buffalo, NY, United States) [13]. We referenced the Gene Ontology (GO) and Kyoto Encyclopedia of Genes and Genomes (KEGG) databases to perform functional annotation on these proteins [14,15]. Additionally, to illustrate their interactions, we utilized GeneMANIA for protein-protein interaction analysis and visualization of the obtained CPPs [16].

Exploration of potential drug molecules

We identified the CPPs with a significant causal impact on MN as potential therapeutic targets. We utilized the Drug Signatures Database (DSigDB) provided by the Enrichr platform to predict potential drugs for the treatment of MN [17]. This platform can screen various small-molecule drugs based on protein or gene targets and assign scores based on the binding affinity between drug molecules and targets. Ultimately, it identifies the most promising drug candidates.

Statistical analysis

We conducted MR analysis using the TwoSampleMR package (version 0.5.6, MRC Integrative Epidemiology Unit, Bristol, United Kingdom) within the R platform (version 4.3.1) [18]. Additionally, we utilized the ggplot package (3.4.3, RStudio/Posit, Boston, MA, United States) to create volcano plots, bubble plots, and bar charts.

## Results

Identification of CPPs causally related to MN

**Figure 1:**
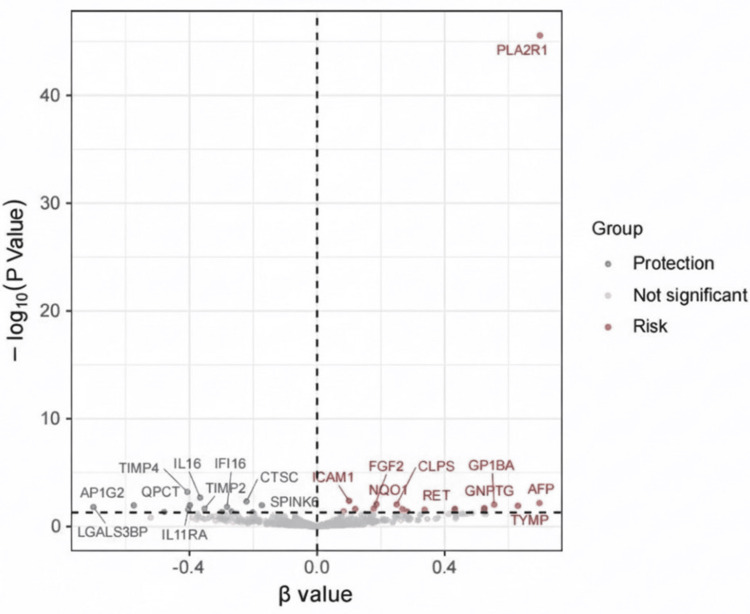
Volcano plot of the results of Mendelian randomization analysis of circulating plasma proteins and MN Red represents a positive association with the risk of MN, and blue represents a negative association. MN: membranous nephropathy

After conducting MR analysis, we screened 30 CPPs out of the initial 734 that exhibited a significant causal relationship with MN. These proteins are as follows: LGALS3BP, AP1G2, BCAN, TIMP4, IL11RA, QPCT, IL16, TIMP2, CD5L, IFI16, PRDX6, CTSC, REG3G, SPINK6, ERAP2, ICAM1, GPC5, FGF2,NQO1, CLPS, LRRC15, FAM3D, B4GALT1, EPHB6, RET, SEMA3C, GNPTG, GP1BA, TYMP, and AFP (Figure 1). Among them, 14 were negatively correlated with the occurrence of MN, while 16 were positively correlated. Except for GPC5, the remaining 29 circulating plasma proteins each had only one available SNP, and thus, the Wald ratio method was employed for analysis. GPC5 had two IVs, so we opted for the IVW method. The results indicated the absence of heterogeneity and pleiotropy.

Functional enrichment

**Figure 2:**
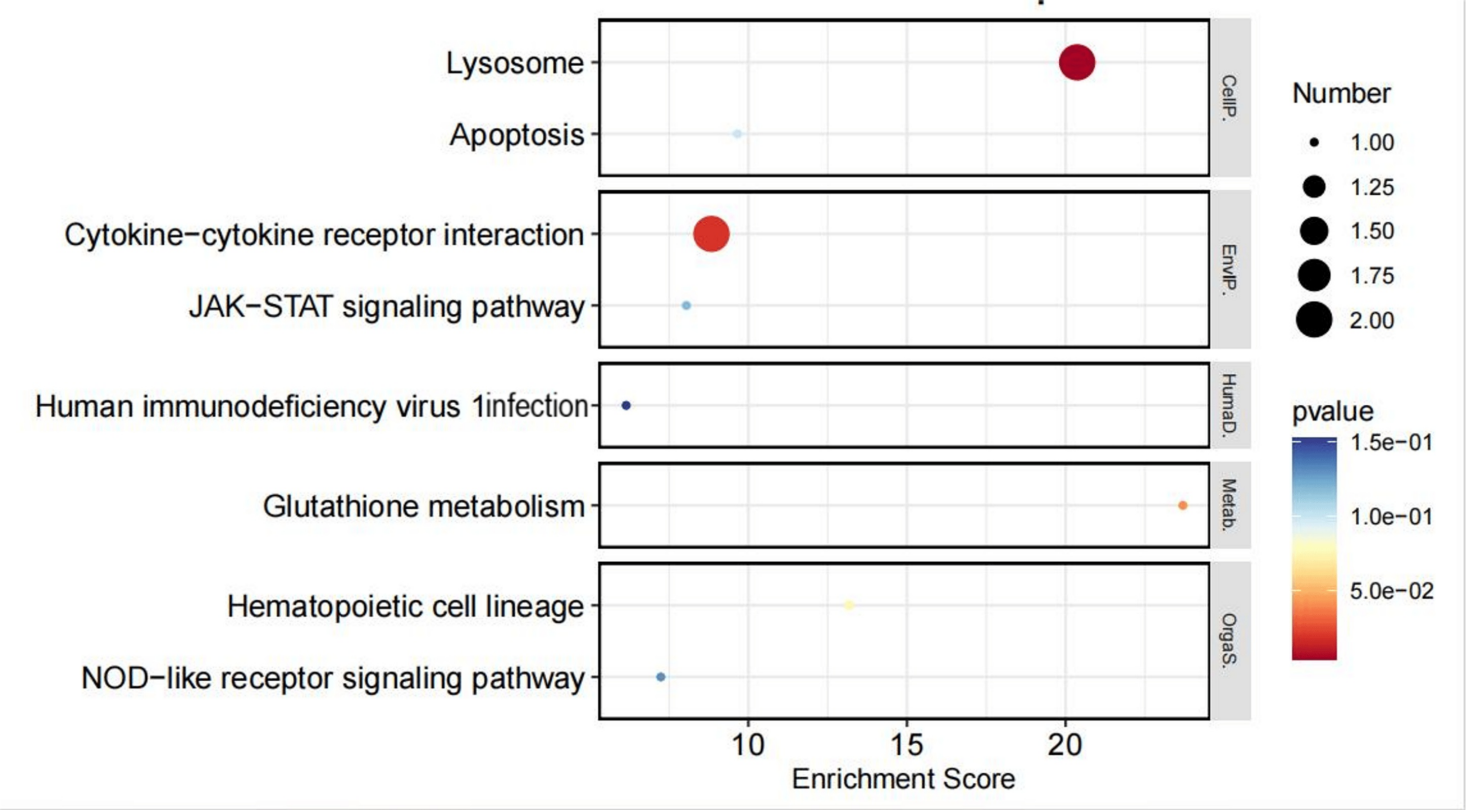
KEGG pathway enrichment analysis of risk-associated CPPs KEGG: Kyoto Encyclopedia of Genes and Genomes; CPP: circulating plasma proteins

**Figure 3:**
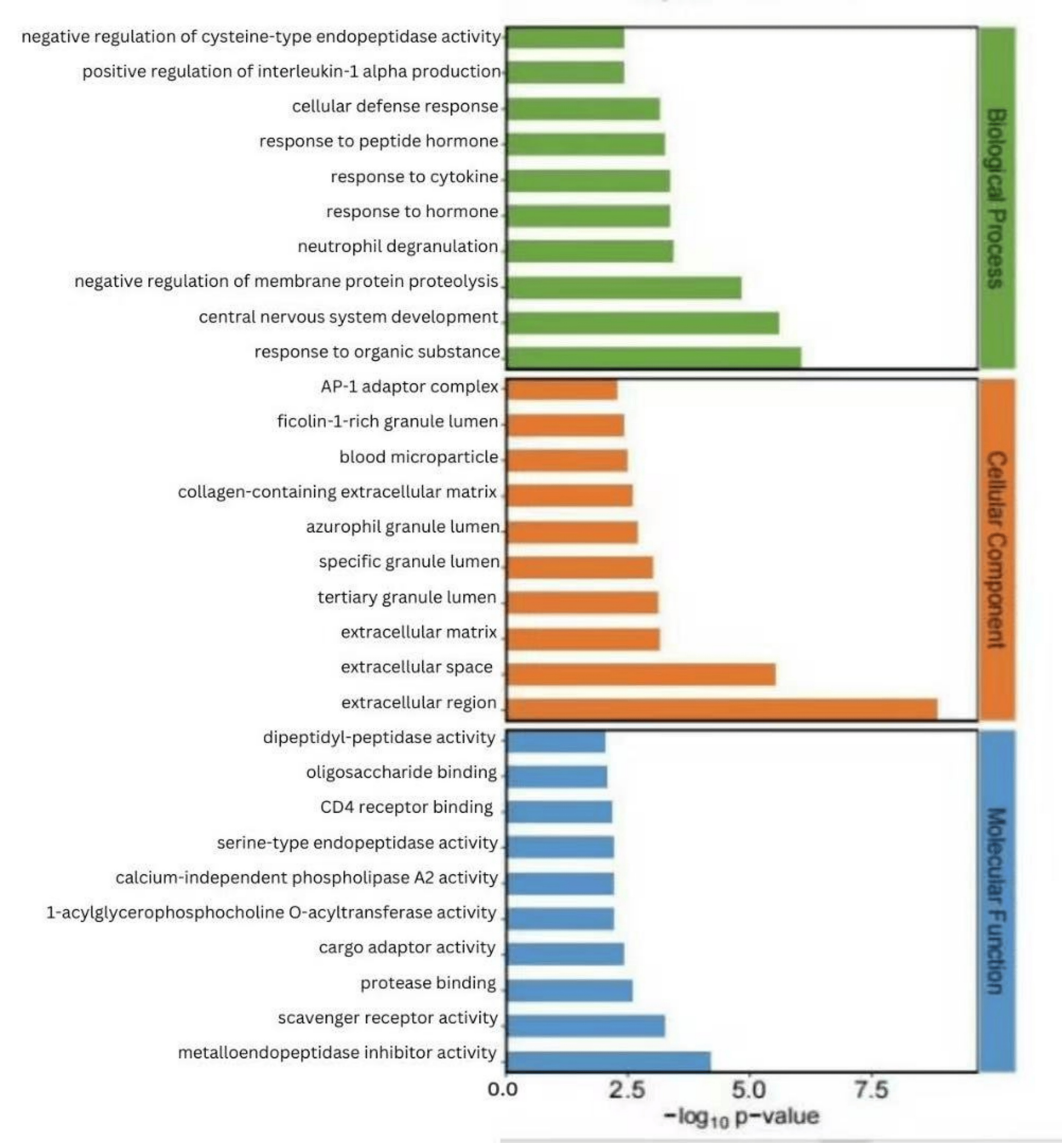
KEGG pathway and GO term enrichment analysis of protective CPPs KEGG: Kyoto Encyclopedia of Genes and Genomes; GO: Gene Ontology; CPP: circulating plasma proteins

**Figure 4:**
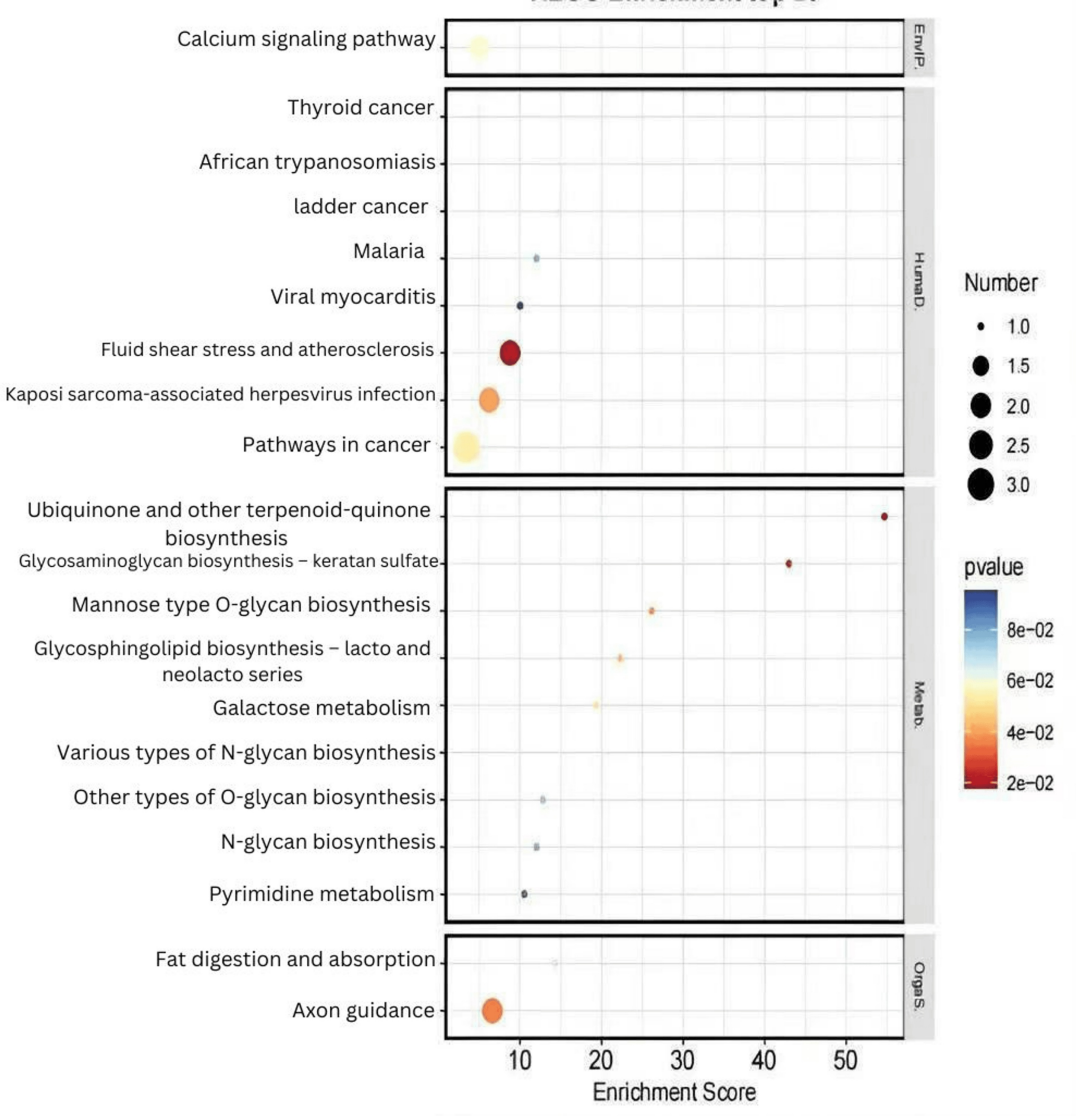
Top 20 significantly enriched KEGG pathways from the enrichment analysis, showing both the enrichment scores and statistical significance of the identified pathways KEGG: Kyoto Encyclopedia of Genes and Genomes

**Figure 5:**
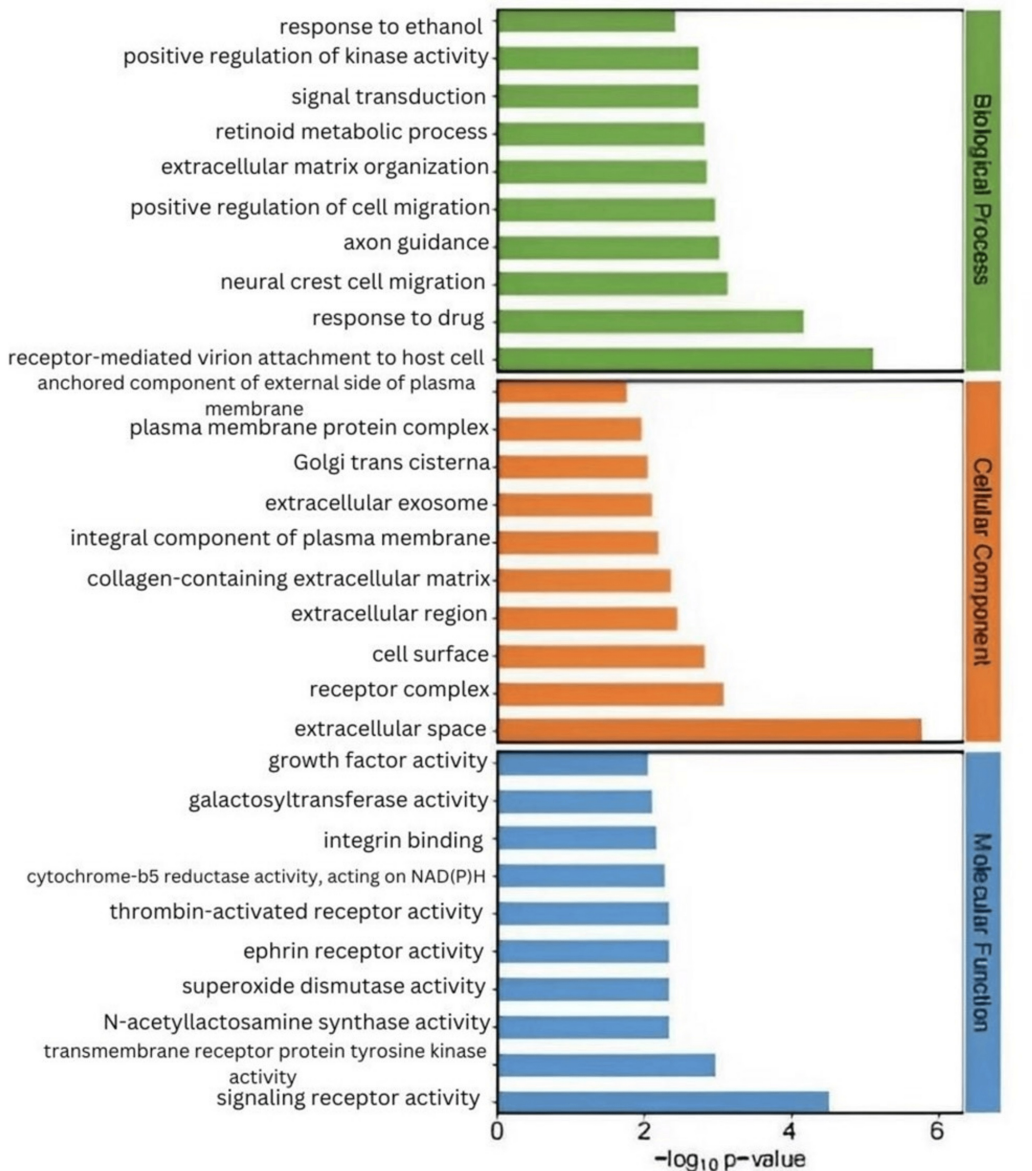
Top 30 significantly enriched gene ontology terms across three ontological categories (biological process, cellular component, and molecular function)

We considered the 16 CPPs positively correlated with MN to be risk factors and the 14 CPPs negatively correlated as protective factors. We conducted separate enrichment analyses for protective and risk factors. Risk factors were primarily involved in several metabolic pathways, such as galactose metabolism, various types of N-glycan biosynthesis, other types of O-glycan biosynthesis, and pyrimidine metabolism, while protective factors were mainly enriched in pathways such as lysosome, glutathione metabolism, the JAK-STAT signaling pathway, and the NOD-like receptor signaling pathway (Figures 2-5).

PPI network

**Figure 6:**
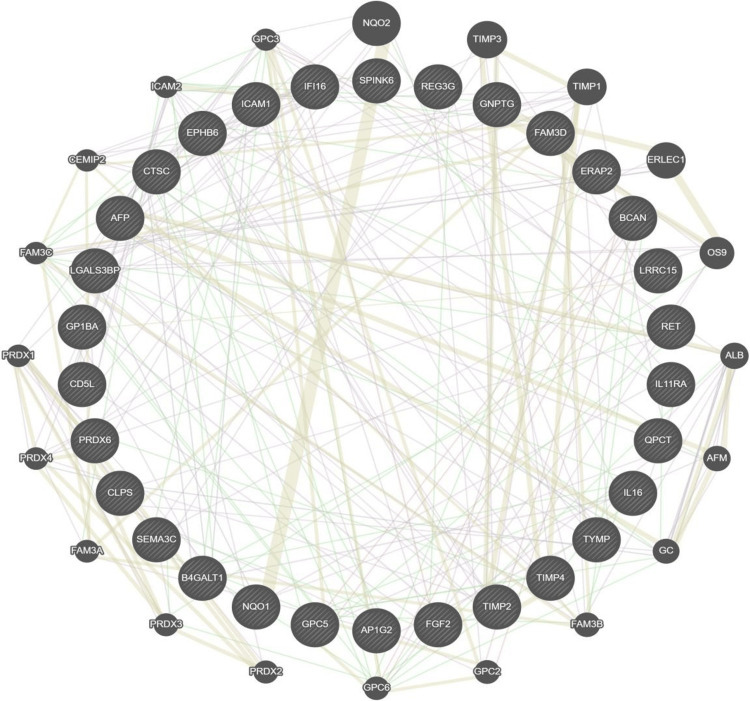
PPI interaction network of significant circulating plasma proteins The line thickness represents the interaction strength, the node size represents the contribution in the network, purple represents co-expression, pink represents physical interactions, yellow represents shared protein domains, blue represents co-localization, and green represents genetic interactions. PPI: protein-protein interaction

To better understand the interactions between the protective factors and risk factors we screened, as well as their associations with other proteins, we constructed a complete PPI network based on the protein interaction data provided in the database. In the network, a larger dot size represents a greater contribution. The core of the PPI network consists of 30 proteins. Secondly, the thickness of the line represents the strength of interaction, and NQO1 and NQO2 have the highest interaction strength. Furthermore, NQO1 was identified as a risk factor for MN (Figure 6).

Identification of a candidate drug molecule

Based on our identification of CPPs that have a causal effect on MN risk, we further identified drugs from the DsigDB database that could target these proteins and be potential therapeutic molecules for MN. After screening by p-value, odds ratio, and target binding score, we found that curcumin could be a potential therapeutic molecule for MN.

## Discussion

MN, as a significant subtype of nephrotic syndrome, exhibits a persistent high prevalence worldwide [19]. As an autoimmune disease, the exact pathogenic mechanisms of MN remain incompletely understood. Current treatment strategies primarily revolve around immunosuppressive therapies, often necessitating the use of corticosteroids and immunosuppressants, which are associated with substantial side effects. Prior research has elucidated the central role of anti-PLA2R antibodies in the pathogenesis of MN, leading to the development of targeted monoclonal antibody therapies for clinical use [20]. However, despite these advancements, the recurrence and progression of MN continue to pose significant challenges. Therefore, there is an urgent need to identify additional therapeutic targets and develop novel targeted drugs with increased efficacy and reduced side effects. This holds great significance for the effective treatment and prevention of MN progression.

In previous studies, numerous genes and proteins associated with MN have been revealed [21]. However, these studies have mainly focused on basic differential and correlation analyses, lacking the ability to establish a clear cause-and-effect relationship. Changes in factors within the study populations have led to less reliable research outcomes. These limitations are inherent in traditional descriptive, observational, and cohort studies. Nonetheless, identifying the factors that contribute to the onset of the disease is crucial for drug development. Therefore, in this study, we chose to employ MR analysis to investigate the causal relationship between circulating plasma proteins and MN using large-scale population-wide summary GWAS data. This approach helped us uncover new therapeutic targets for MN. By integrating pQTL loci summarized from previous studies, we investigated CPPs causally linked to the development of MN. Our findings revealed that 14 proteins were significantly inversely correlated with the onset of MN, indicating their potential protective roles. Conversely, 16 proteins showed a positive correlation, suggesting they may act as potential risk factors for MN. These results effectively eliminate the confounding influences of prospective study factors. They provide a clear genetic-level delineation of the relationship between these proteins and MN, with no significant heterogeneity observed. Among the proteins identified, several have been previously implicated in the etiology of MN. SERPINA1, for instance, is known for its roles in autoimmune processes [22], which align with our findings that suggest a positive correlation with MN progression. These proteins could contribute to the pathogenic landscape by modulating immune responses or by altering the coagulation cascade, potentially exacerbating the disease process. Conversely, proteins such as IL16 are shown to be negatively correlated with MN, suggesting a potential protective effect. This effect may be mediated through the suppression of inflammatory pathways or by promoting tissue repair and remodeling processes [23]. Similarly, the addition of other negatively correlated proteins like CST3, which plays a role in inhibiting cysteine protease activity [24], and SPP1, involved in biomineralization and inflammation, further suggests a complex network of protective mechanisms against MN [25]. In the context of positively correlated proteins, GP1BA and FGG indicate a possible contribution to the thrombotic processes associated with MN [26], while RET involvement hints at a role for endocrine and growth factor dysregulation in disease manifestation [27]. The oxidative stress marker NQO1 also emerged as a protein with positive correlation, suggesting an exacerbating role in MN pathophysiology through inflammatory pathways [28]. It is noteworthy that these findings not only reinforce the established connections but also propose additional proteins that could be instrumental in MN progression or protection.

The presence of ADIPOR1, associated with anti-inflammatory functions, adds to the protective profile [29]. Subsequent to the identification of proteins correlated with the onset of MN, we constructed a PPI network to visualize the relationships among these proteins. The 30 CPPs associated with MN are not isolated; instead, they exhibit complex relationships characterized by co-expression and mutual interactions. Further enrichment analysis revealed that the 16 plasma proteins positively correlated with MN likely represent risk factors, whereas those negatively correlated may act as protective factors. We conducted separate enrichment analyses for these protective and risk factors. In the context of this study, the enrichment of risk factors in metabolic pathways is consistent with the literature, suggesting that disruptions in carbohydrate metabolism may contribute to the development of MN through the accumulation of glycated end products and oxidative stress, which can impair renal function [30]. The association with pyrimidine metabolism pathways may reflect the increased cellular turnover and DNA repair processes that occur in response to tissue damage in MN. Conversely, the protective factors' enrichment in the lysosomal pathway supports the hypothesis that efficient degradation of immune complexes, which are often implicated in MN, may mitigate disease progression. Additionally, the role of glutathione metabolism in maintaining redox homeostasis could explain its protective association, as oxidative stress is a known contributor to MN pathology. The JAK-STAT and NOD-like receptor signaling pathways have been linked to anti-inflammatory and immunomodulatory responses, suggesting that modulation of these pathways might underlie the protective effects observed [31,32].

Intriguingly, through our analysis, curcumin emerges as a potential therapeutic molecule for treating MN. Curcumin is renowned for its antioxidant properties, immune modulation capabilities, and anti-inflammatory actions. These features of curcumin suggest its suitability as a therapeutic agent, particularly in diseases where oxidative stress and inflammatory responses play a pivotal role.

Study limitations

While this study uses MR to suggest causal directions, several limitations must be noted. Our findings are derived from European-ancestry data, limiting generalizability. The validity of MR hinges on the assumption of no pleiotropy, which cannot be fully verified; weak instruments or undetected pleiotropy may bias results. Importantly, the absence of a multiple-testing correction across 734 proteins increases the risk of false positives, and the heavy reliance on single-SNP instruments for many proteins restricts our ability to perform rigorous pleiotropy-robust sensitivity analyses. This reduces the robustness of causal claims for individual targets. Furthermore, the use of summary-level data prevented patient-stratified analysis. Finally, the nomination of curcumin as a therapeutic agent remains a bioinformatic prediction requiring direct experimental and clinical validation.

## Conclusions

This MR study identified 30 circulating proteins causally linked to MN, including several that align with known immunopathogenic pathways and current therapies such as calcineurin inhibitors, rituximab, and glucocorticoids. Notably, proteins related to B-cell regulation and immune activation showed concordance with mechanisms targeted by these treatments and with the biology underlying circulating PLA2R levels. We also identified protective proteins and pathways involving oxidative stress and inflammation, and drug-repositioning analysis highlighted curcumin as a potential immunomodulatory candidate. These findings strengthen biological understanding of MN and suggest several proteins as promising therapeutic targets, warranting further mechanistic and translational investigation.
